# Supplementary material for: An Overview of Angiostrongylus cantonensis (Nematoda: Angiostrongylidae), an Emerging Cause of Human Angiostrongylosis on the Indian Subcontinent
Source: Pathogens. 2023 Jun 20;12(6):851. doi: 10.3390/pathogens12060851 (PMC10304679; doi:10.3390/pathogens12060851)
Supplement: Supplementary file 1 [file pathogens-12-00851-s001.zip › pathogens-2348880-supplementary.pdf]

Supplementary files

Table S1. List of Indian states where eating snail meat is common.

Family	Species	Places	Preparation	References
Ampullariidae	*Pila globosa*	CH, MP, NE, OR, TN, WB	Cooked	[136,138,142,166,187–191]
Ampullariidae	*Pila scutata*	BR, NE, WB	Cooked	[138,142]
Ampullariidae	*Pila theobaldi*	NE, WB	Cooked	[142]
Ampullariidae	*Pila virens*	NE, WB	Cooked	[142]
Ariophantidae	*Cryptozona bistrialis*	NE, WB	Cooked	[136,140,143]
Lymnaeinae	*Lymnaea* sp.	NE	Cooked	[192,193]
Pachychilidae	*Brotia costua*	WB	Cooked	[139]
Paludomidae	*Paludomus* sp.	NE	Cooked	[192]
Planorbidae	*Anisus convexiusculus*	NE, WB	Cooked	[140,143]
Viviparidae	*Angulyagra* sp.	NE	Cooked	[194]
Viviparidae	*Bellamya bengalensis*	JK, NE, WB	Cooked	[139,142,143,195]
Viviparidae	*Bellamya dissimilis*	NE	Cooked	[136,191]
Viviparidae	*Bellamya* sp.	BR	Cooked	[138]
Viviparidae	*Cipangopaludina lecythis*	NE	Cooked	[194]
Viviparidae	*Viviparus viviparus*	BR	Cooked	[196]
Viviparidae	*Viviparus bengalensis*	BR	Cooked	[196]

State Abbreviations: BR — Bihar, CH– Chandigarh, JK — Jharkhand; KL — Kerala, MP — Madhya Pradesh, OR — Orissa, TN — Tamil Nadu, WB — West Bengal. NE — Northeast India includes (AR — Arunachal Pradesh, AS — Assam, NL — Nagaland, MZ–Mizoram, SK — Sikkim, and T — Tirupura), where eating snails is more common.

Table S2. Overview of snakes are used for zootherapy in tribal communities.

Family	Species	Location	Application	References
Colubridae	*Dendrelaphis tristis*	NE*	Ethnomedicine	[197]
Colubridae	*Ptyas mucosa*	NE*, MH, TN	Ethnomedicine	[159,198]
Elapidae	*Naja naja*	NE*, RJ, TN	Ethnomedicine	[161,162,190,199]
Elapidae	*Ophiophagus hannah*	NE*, TN	Ethnomedicine	[162,197]
Pythonidae	*Python reticulatus*	NE*, TN	Ethnomedicine	[162,197,200]
Viperidae	*Daboia* sp.	NE*	Ethnomedicine	[192]
Viperidae	*Echis coloratus*	NE*	Ethnomedicine	[164]

State abbreviations: MH — Maharashtra, RJ – Rajasthan, TN — Tamil Nadu, NE — Northeast India (AR — Arunachal Pradesh, AS — Assam, NL — Nagaland, MZ — Mizoram, SK — Sikkim, T — Tripura). NE* — Northeastern India (Indigenous peoples use snakes in the practice of traditional medicine).

Table S3. Overview of where frog meat is common.

Family	species	Location	Preparation	Reference
Bufonidae	*Duttaphrynus melanostictus*	MZ	Cooked	[174]
Dicroglossidae	*Euphlyctis cyanophlyctis,*	AS, NL, JK	Cooked	[176,201–203]
Dicroglossidae	*Euphlyctis hexadactylus,*	AS	Cooked	[204]
Dicroglossidae	*Euphlyctis ghoshi*	NL	Cooked	[204]
Dicroglossidae	*Fejervarya limnocharis*	JK, NL	Cooked	[176,202]
Dicroglossidae	*Fejervarya teraiensis*	NL	Cooked	[175,176]
Dicroglossidae	*Hoplobatrachus crassus*	AS, NL	Cooked	[175]
Dicroglossidae	*Hoplobatrachus litoralis*	NL	Cooked	[176,204]
Dicroglossidae	*Hoplobatrachus rugulosus*	NL	Cooked	[175,176]
Dicroglossidae	*Hoplobatrachus tigerinus,*	AS, NL, WB	Cooked	[175,176]
Dicroglossidae	*Nanorana liebigii*	AS, NL	Cooked	[176,204]
Dicroglossidae	*Phrynoderma karaavali*	TN, KL	Cooked	[205–209]
Megophryidae	*Megophrys major*	NL	Cooked	[175,176]
Megophryidae	*Megophrys flavipunctata*	NL	Cooked	[175,176]
Ranidae	*Amolops Himalayanu*	MZ, SK	Cooked	[174,210]
Ranidae	*Amolops indoburmanensis*	MZ	Cooked	[174]
Ranidae	*Amolops marmoratus*	NL	Cooked	[175,176]
Ranidae	*Odorrana chloronota*	MZ	Cooked	[174]
Ranidae	*Pterorana Khare*	NL	Cooked	[175,176]
Ranidae	*Rana Tigrina*	AP, UK, MP	Cooked	[161,201,211]
Ranidae	*Sylvirana lacrima*	MZ	Cooked	[174]
Rhacophoridae	*Polypedates teraiensis*	NL	Cooked	[176,202]
Rhacophoridae	*Polypedates leucomystax*	AS	Cooked	[204]
Rhacophoridae	*Rhacophorus bipunctatus*	NL	Cooked	[175,176]
Rhacophoridae	*Rhacophorus maximus*	NL	Cooked	[175,176]
Rhacophoridae	*Zhangixalus smaragdinus*	MZ	Cooked	[174]

State abbreviation; AR — Arunachal Pradesh, AS — Assam, JK — Jharkhand; KL — Kerala, NL — Nagaland, MP — Madhya Pradesh. MZ — Mizoram, SK — Sikkim, UK — Uttarakhand, WB — West Bengal, TN — Tamil Nadu.
